# Supplementary material for: Different Responses of Soil Bacterial and Fungal Communities in Three Typical Vegetations following Nitrogen Deposition in an Arid Desert
Source: Microorganisms. 2023 Oct 1;11(10):2471. doi: 10.3390/microorganisms11102471 (PMC10609353; doi:10.3390/microorganisms11102471)
Supplement: Supplementary file 1 [file microorganisms-11-02471-s001.zip › microorganisms-2608444-supplementary.pdf]

**Table S1** Mantel test analysis between community composition of soil bacteria and fungi and soil physicochemical properties in three plant communities.

| | Variable | *A. sparsifolia* | | *K. caspia* | | Mixing | |
|---|---|---|---|---|---|---|---|
| | | Statistic | Signif | Statistic | Signif | Statistic | Signif |
| Bacteria | SOC | −0.092 | 0.695 | −0.079 | 0.701 | 0.136 | 0.168 |
| | pH | −0.226 | 0.964 | −0.117 | 0.802 | **0.308** | **0.011** |
| | AP | −0.086 | 0.712 | −0.154 | 0.863 | 0.212 | 0.095 |
| | AK | 0.172 | 0.108 | −0.074 | 0.629 | **0.329** | **0.024** |
| | TN | −0.120 | 0.751 | 0.271 | 0.072 | −0.104 | 0.721 |
| | $NO_3^-$−N | 0.271 | 0.071 | −0.119 | 0.710 | **0.331** | **0.014** |
| | $NH_4^+$−N | 0.147 | 0.234 | 0.038 | 0.315 | 0.171 | 0.117 |
| | AN | 0.067 | 0.378 | −0.072 | 0.591 | 0.184 | 0.061 |
| Fungi | SOC | −0.082 | 0.687 | 0.057 | 0.301 | −0.028 | 0.558 |
| | pH | −0.088 | 0.715 | 0.154 | 0.138 | 0.082 | 0.247 |
| | AP | 0.182 | 0.092 | −0.014 | 0.485 | 0.145 | 0.201 |
| | AK | 0.134 | 0.181 | −0.151 | 0.815 | 0.205 | 0.140 |
| | TN | 0.078 | 0.297 | **0.254** | **0.047** | −0.302 | 0.944 |
| | $NO_3^-$−N | 0.260 | 0.090 | −0.119 | 0.740 | −0.010 | 0.528 |
| | $NH_4^+$−N | **0.497** | **0.009** | 0.136 | 0.211 | 0.109 | 0.251 |
| | AN | 0.078 | 0.321 | −0.074 | 0.645 | −0.001 | 0.500 |

**Note:** SOC, soil organic carbon; AP, available phosphorous; AK, available potassium; TN, total nitrogen; $NO_3^-$ −N, nitrate; $NH_4^+$ −N, ammonium; AN, available nitrogen. This result was conducted with 999 permutations.

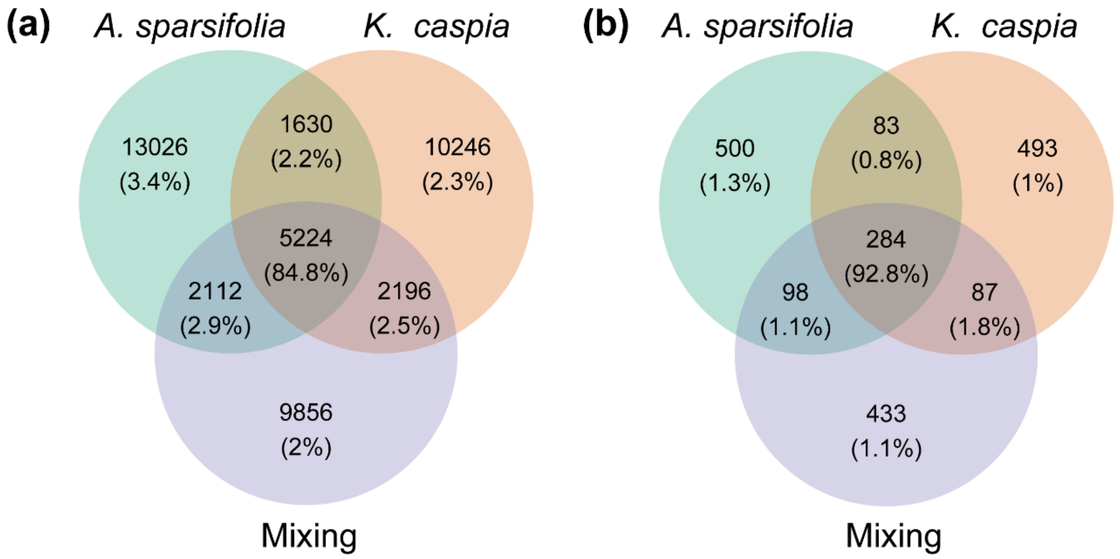


**Fig. S1** Venn diagram shows the number (percentage) bacterial **(a)** and fungal **(b)** ASVs specific to and common to the three plant community types.

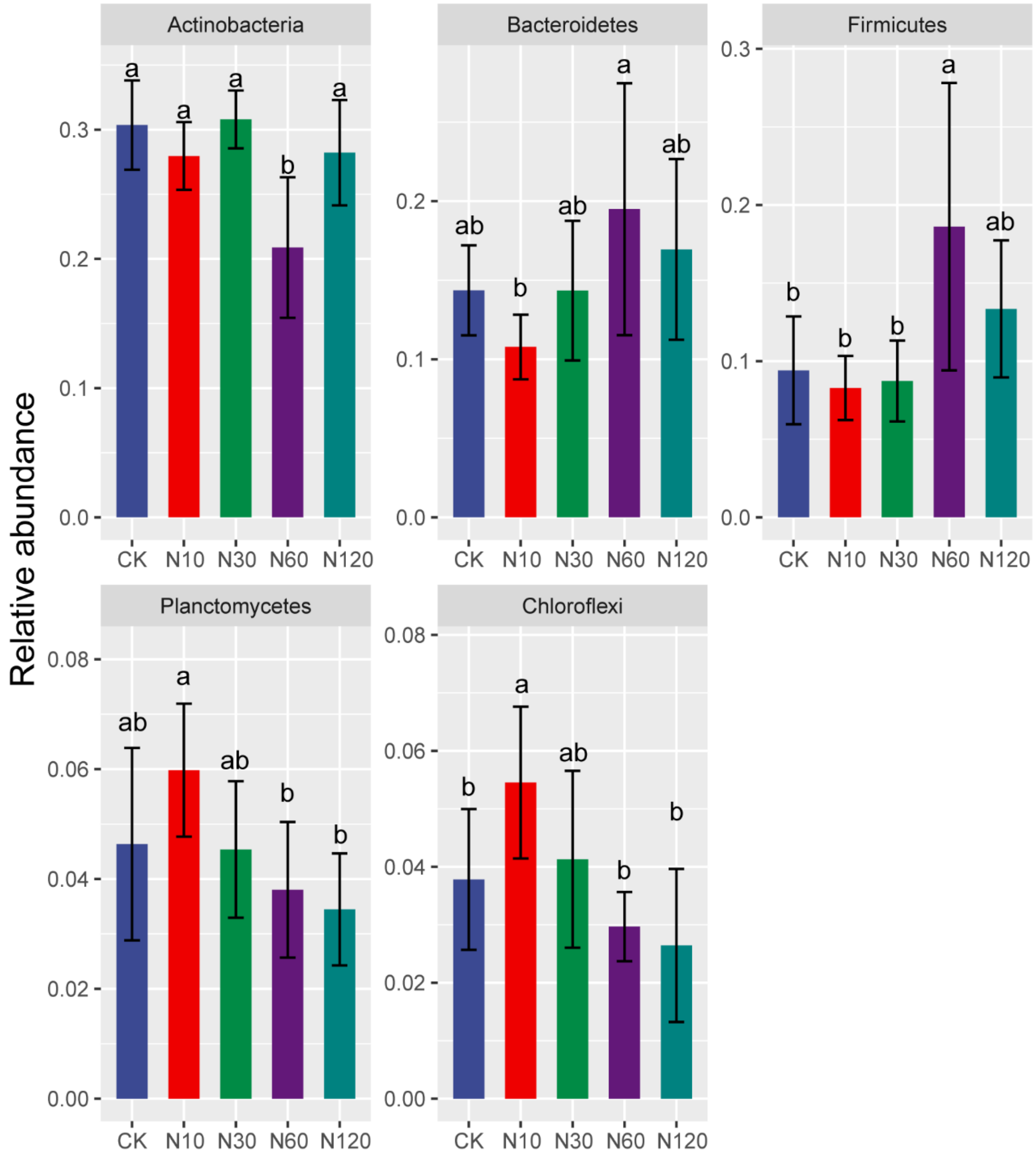


**Fig. S2** Phyla significantly affected by N treatment (*P*<0.05). Different lowercase letters indicate a significant difference among different N addition treatments.

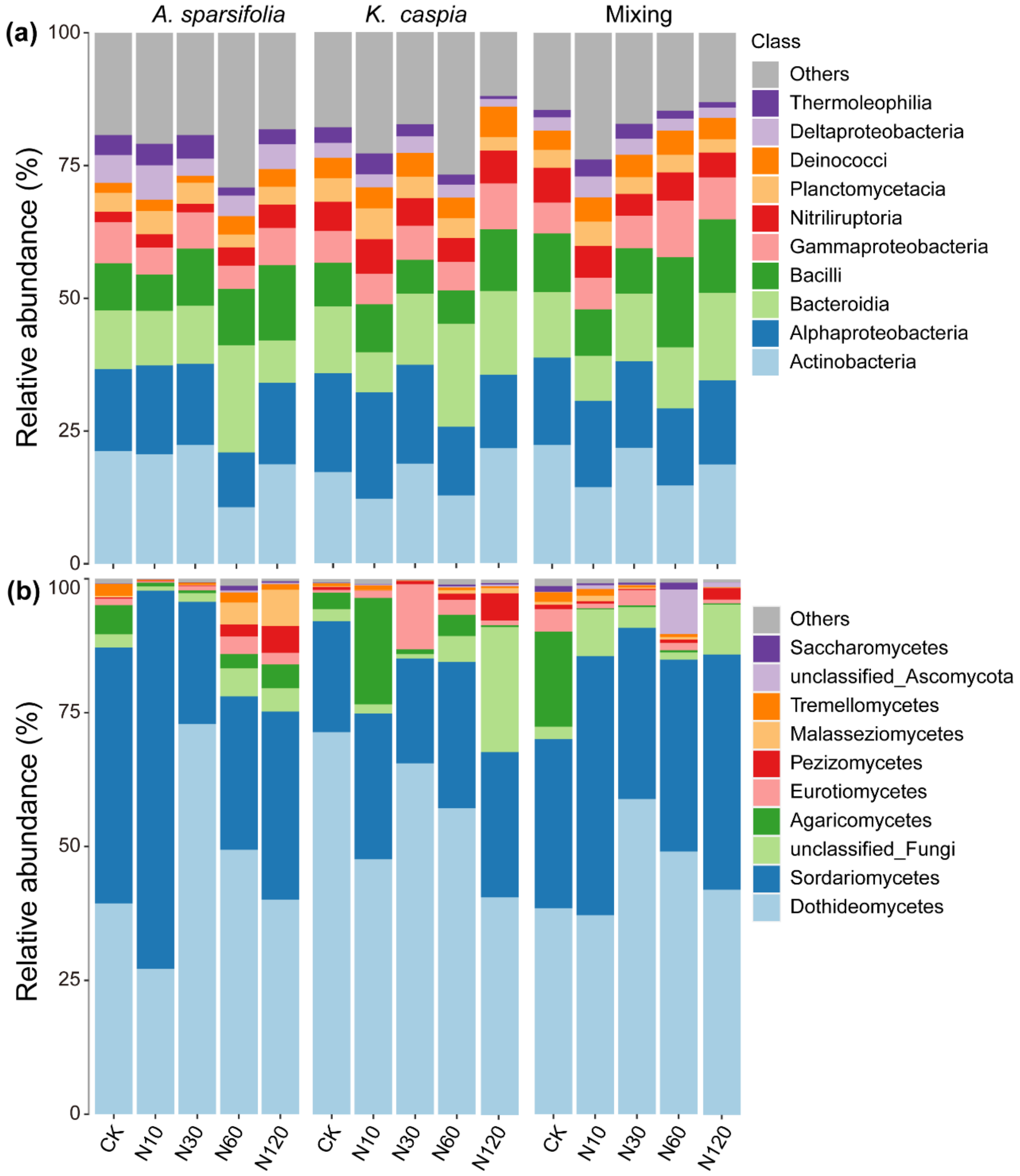


**Fig. S3** Community composition of bacteria **(a)** and fungi **(b)** at class level (top 10).

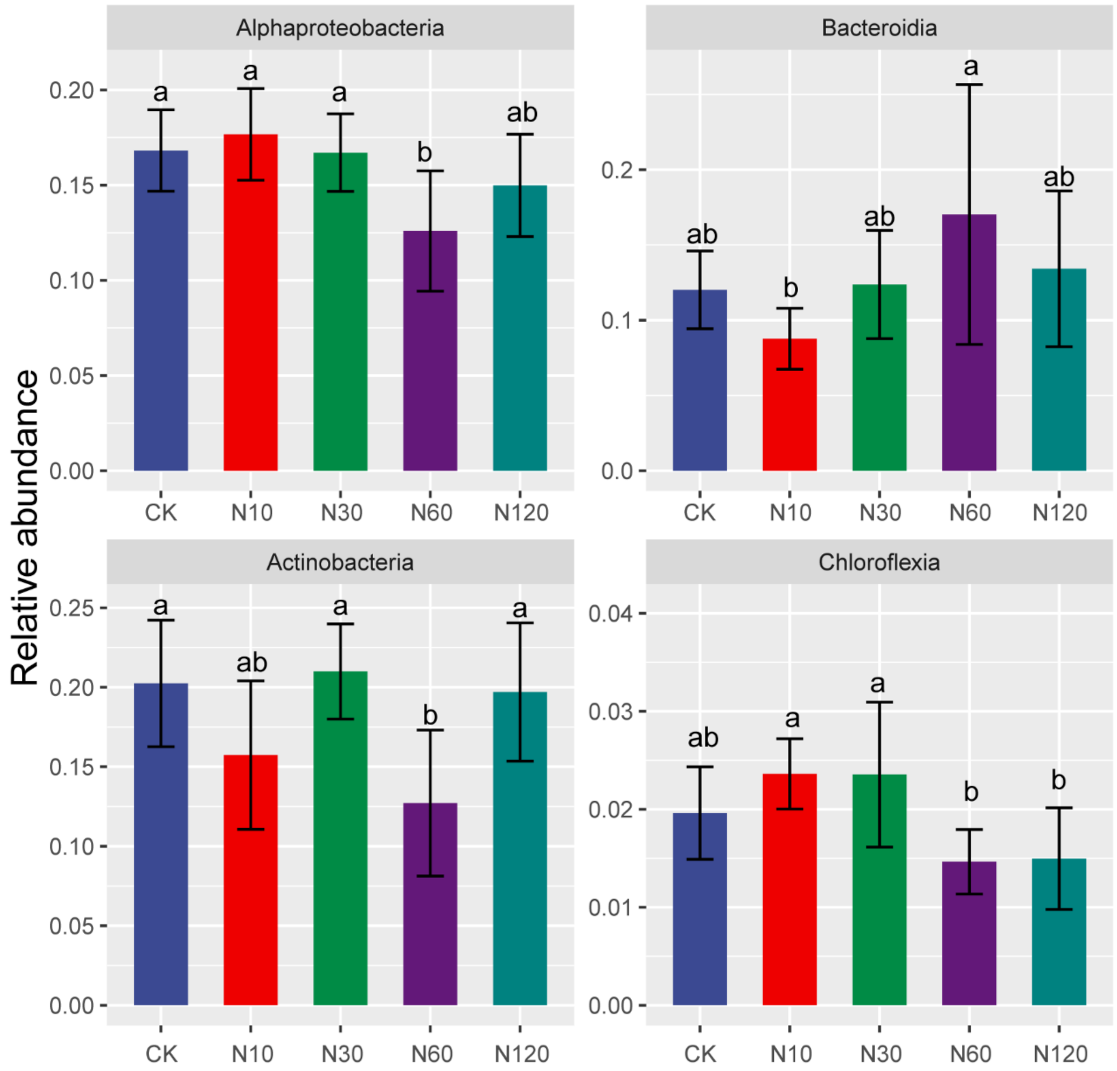


**Fig. S4** The classes significantly affected by N treatment ($P$<0.05). Different lowercase letters indicate a significant difference among different N addition treatments.

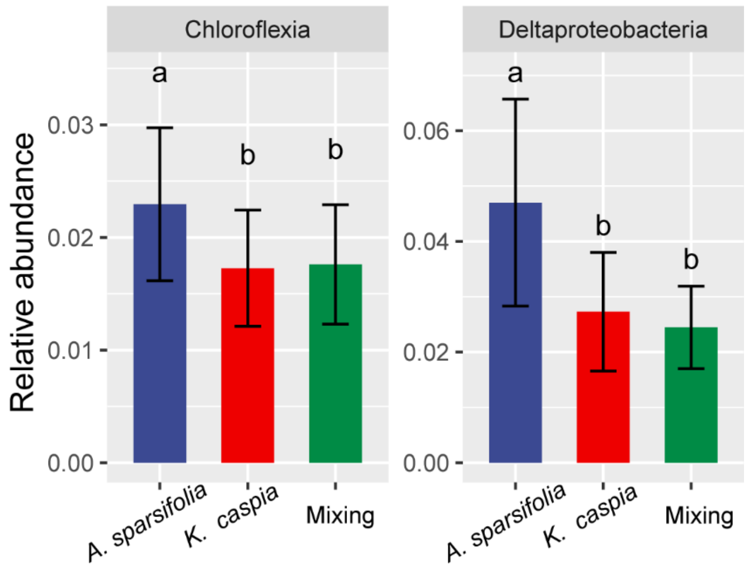


**Fig. S5** The classes significantly affected by plant community type ($P$<0.05). Different lowercase letters indicate a significant difference among different N addition treatments.

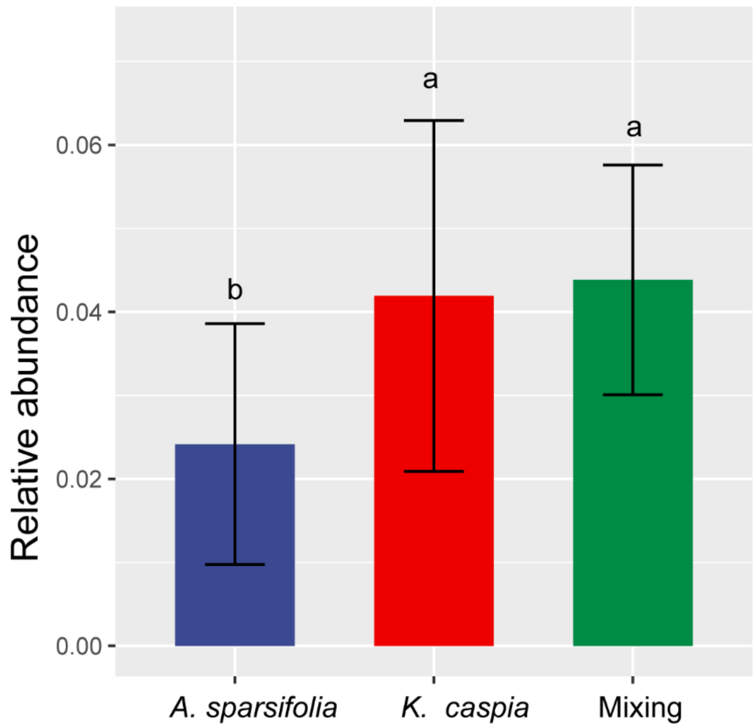


**Fig. S6** The relative abundance of Deinococcus-Thermus. Different lowercase letters indicate a significant difference among different plant communities.

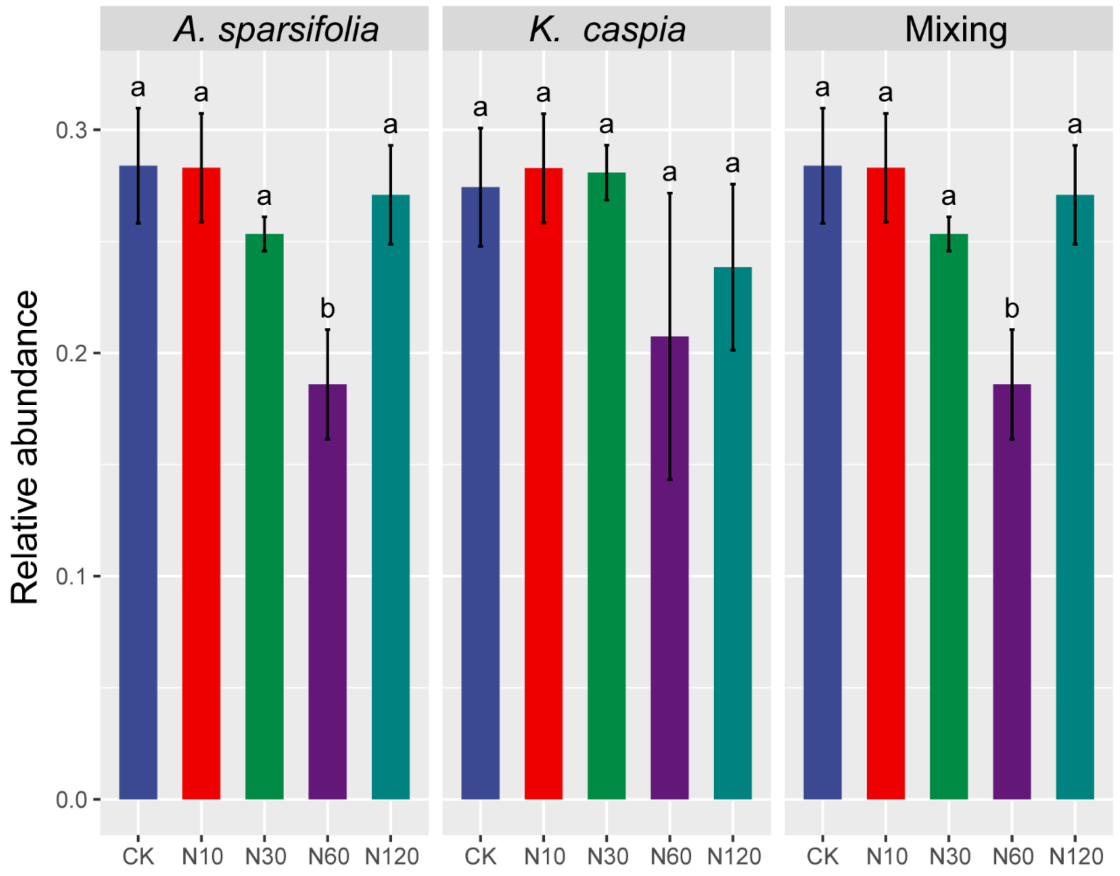


**Fig. S7** The relative abundance of Proteobacteria. Different lowercase letters indicate a significant difference among different plant communities.

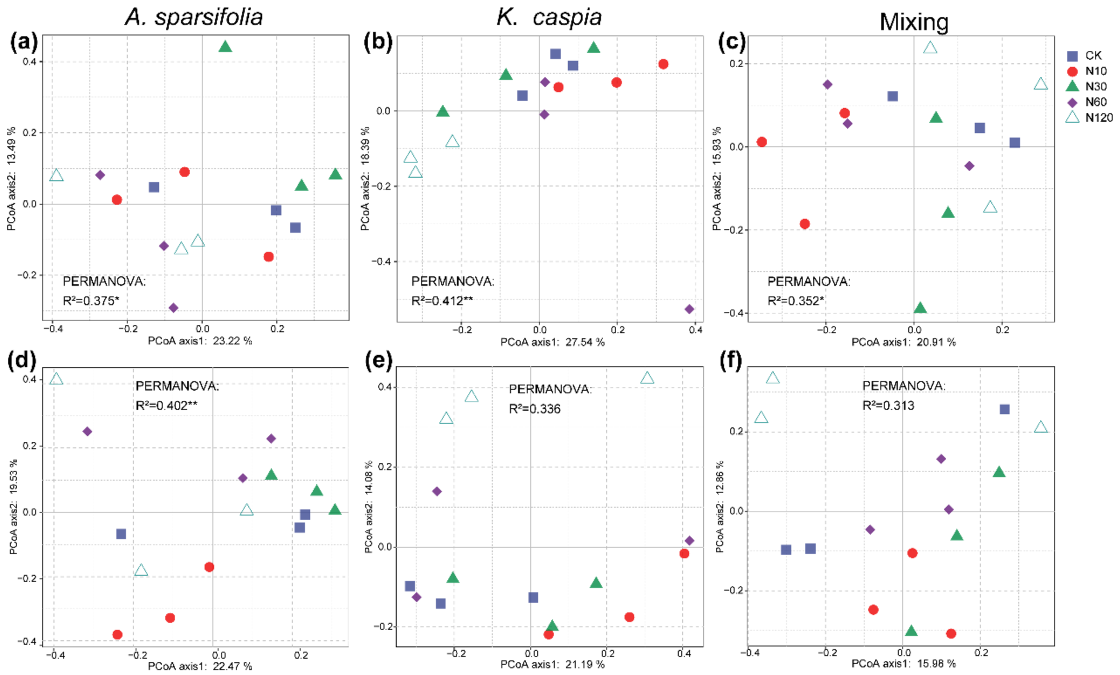


**Figure S8** Principal coordinate analysis (PCoA) of the bacterial **(a, b, c)** and fungal **(d, e, f)** communities based on the Bray-Curtis distance of ASV. Permutational analysis of variance (PERMANOVA) shows the effects of N addition treatment on the Bray-Curtis distance of ASVs.

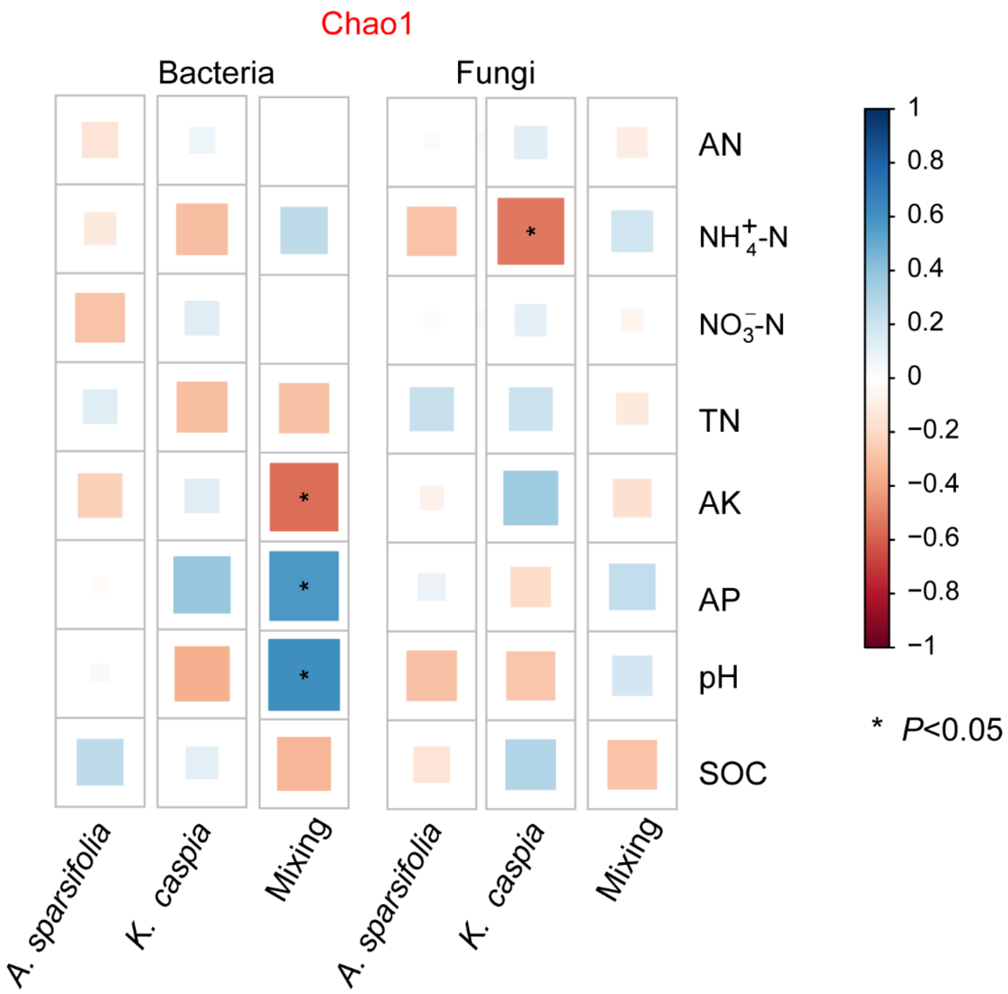


**Figure S9** Pearson correlation between α-diversity (Chao1) of bacterial **(a)** and fungal **(b)** communities and soil properties. Asterisks indicate significant relationships ($P$<0.05). SOC, soil organic carbon; AP, available phosphorus; AK, available potassium; TN, total nitrogen; $NO_3^-$−N, nitrate; $NH_4^+$−N, ammonium; AN, available nitrogen.

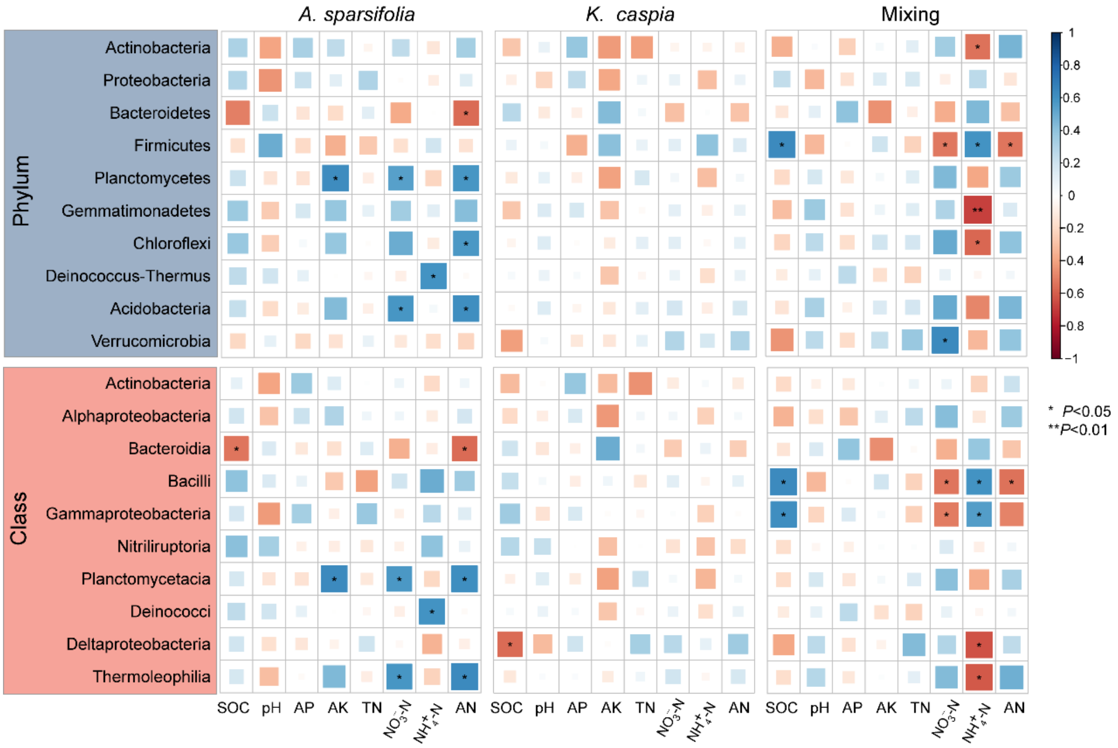


**Fig. S10** Pearson correlation between the relative abundance of bacterial dominant phyla and classes and soil properties. Asterisks indicate significant relationships. SOC, soil organic carbon; AP, available phosphorus; AK, available potassium; TN, total nitrogen; $NO_3^-$ −N, nitrate; $NH_4^+$ −N, ammonium; AN, available nitrogen.

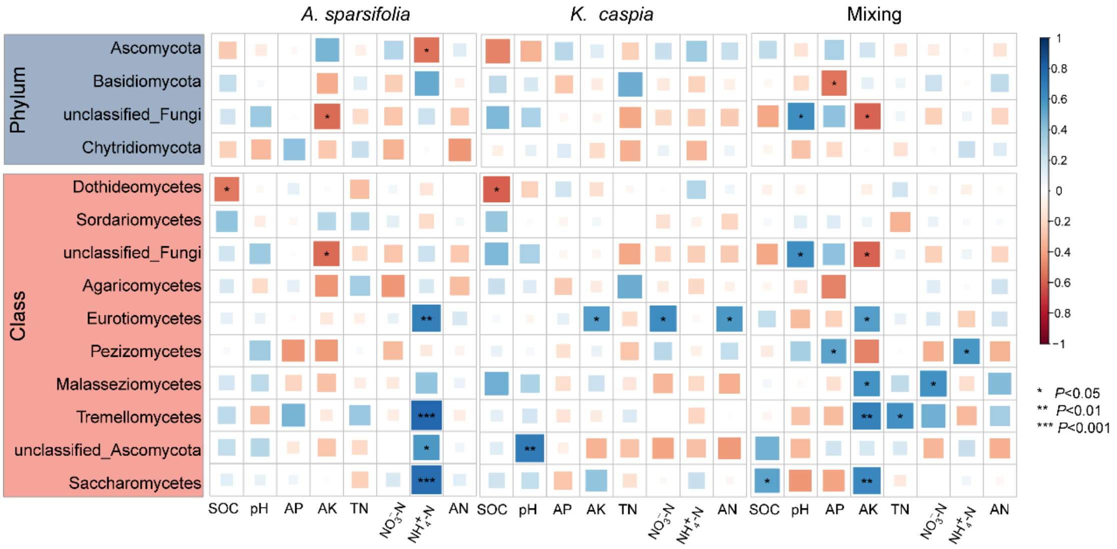


**Figure S11** Pearson correlation between the relative abundance of fungal dominant phyla and classes and soil properties. Asterisks indicate significant relationships. SOC, soil organic carbon; AP, available phosphorus; AK, available potassium; TN, total nitrogen; $NO_3^-$ −N, nitrate; $NH_4^+$ −N, ammonium; AN, available nitrogen.
